# Supplementary material for: FastqPuri: high-performance preprocessing of RNA-seq data
Source: BMC Bioinformatics. 2019 May 3;20:226. doi: 10.1186/s12859-019-2799-0 (PMC6500068; doi:10.1186/s12859-019-2799-0)
Supplement: Supplementary file 2 — Archive of FastqPuri. Archive containing all files needed to install and run FastqPuri v1.0.6. Date stamp March 22, 2019. (GZ 47,819 kb) [file 12859_2019_2799_MOESM2_ESM.gz › FastqPuri-1.0.6/html/trim_8h.html]

FastqPuri: include/trim.h File Reference


FastqPuri


- include

Functions

trim.h File Reference

trims/filter sequences after Quality, N's contaminations.
More...

`#include <stdio.h>`  
`#include "Lmer.h"`  
`#include "fq_read.h"`  
`#include "defines.h"`  
`#include "tree.h"`  
`#include "bloom.h"`  
`#include "adapters.h"`

Include dependency graph for trim.h:

This graph shows which files directly or indirectly include this file:

Go to the source code of this file.

|  |  |
| --- | --- |
| Functions | |
| int | trim\_adapter (Fq\_read \*seq, Ad\_seq \*adap\_list) |
|  | trims sequence based on presence of N nucleotides More... |
|  | |
| int | trim\_sequenceN (Fq\_read \*seq) |
|  | trims sequence based on presence of N nucleotides More... |
|  | |
| int | trim\_sequenceQ (Fq\_read \*seq) |
|  | trims sequence based on lowQ base callings More... |
|  | |
| bool | is\_read\_inTree (Tree \*tree\_ptr, Fq\_read \*seq) |
|  | check if Lread is contained in tree. It computes the score for the read and its reverse complement; if one ot them exceeds the user selected threshold, it returns true. Otherwise, it returns false. More... |
|  | |
| bool | is\_read\_inBloom (Bfilter \*tree\_ptr, Fq\_read \*seq, Bfkmer \*ptr\_Bfkmer) |
|  | checks if a read is in Bloom filter. It computes the score for the read and returns true if it exceeds the user selected threshold. Returns false othersise. More... |
|  | |
| int | Qtrim\_global (Fq\_read \*seq, int left, int right, char type) |
|  | trims left from the left and right from the right More... |
|  | |

## Detailed Description

trims/filter sequences after Quality, N's contaminations.

Author
:   Paula Perez paula.nosp@m.pere.nosp@m.zrubi.nosp@m.o@gm.nosp@m.ail.c.nosp@m.om

Date
:   24.08.2017

## Function Documentation

## ◆ is\_read\_inBloom()

|  |  |  |  |
| --- | --- | --- | --- |
| bool is\_read\_inBloom | ( | Bfilter \* | *ptr\_bf*, |
|  |  | Fq\_read \* | *seq*, |
|  |  | Bfkmer \* | *ptr\_bfkmer* |
|  | ) |  |  |

checks if a read is in Bloom filter. It computes the score for the read and returns true if it exceeds the user selected threshold. Returns false othersise.

Parameters
:   |  |  |
    | --- | --- |
    | ptr\_bf | pointer to Bfilter |
    | seq | fastq read |
    | ptr\_bfkmer | pointer to Procs\_kmer structure (will store global) |

Returns
:   true if read was found, false otherwise

## ◆ is\_read\_inTree()

|  |  |  |  |
| --- | --- | --- | --- |
| bool is\_read\_inTree | ( | Tree \* | *tree\_ptr*, |
|  |  | Fq\_read \* | *seq* |
|  | ) |  |  |

check if Lread is contained in tree. It computes the score for the read and its reverse complement; if one ot them exceeds the user selected threshold, it returns true. Otherwise, it returns false.

Parameters
:   |  |  |
    | --- | --- |
    | tree\_ptr | pointer to Tree structure |
    | seq | fastq read |

Returns
:   true if read was found, false otherwise

## ◆ Qtrim\_global()

|  |  |  |  |
| --- | --- | --- | --- |
| int Qtrim\_global | ( | Fq\_read \* | *seq*, |
|  |  | int | *left*, |
|  |  | int | *right*, |
|  |  | char | *type* |
|  | ) |  |  |

trims left from the left and right from the right

Parameters
:   |  |  |
    | --- | --- |
    | seq | fastq read |
    | left | number of nucleotides to be trimmed from the left |
    | right | number of nucleotides to be trimmed from the right |
    | type | char indicating the type of trimming (Q,A). |

Returns
:   2, since they are all accepted and trim

## ◆ trim\_adapter()

|  |  |  |  |
| --- | --- | --- | --- |
| int trim\_adapter | ( | Fq\_read \* | *seq*, |
|  |  | Ad\_seq \* | *adap\_list* |
|  | ) |  |  |

trims sequence based on presence of N nucleotides

if (adapter length < 16) -> search for seeds 8 nucleotides long else -> search for seeds 16 nucleotides long if (seed found) -> calculate score if score > threshold -> aligner found, trim / discard and exit. else -> search for seeds 8 nucleotides long

Parameters
:   |  |  |
    | --- | --- |
    | seq | pointer to **Fq\_read** |
    | adap\_list | array of **Ad\_seq** |

Returns
:   -1 error, 0 discarded, 1 accepted as is, 2 accepted and trimmed

Note
:   Global input parameters from par\_TF are also used

## ◆ trim\_sequenceN()

|  |  |  |  |  |  |
| --- | --- | --- | --- | --- | --- |
| int trim\_sequenceN | ( | Fq\_read \* | *seq* | ) |  |

trims sequence based on presence of N nucleotides

Parameters
:   |  |  |
    | --- | --- |
    | seq | fastq read |

Returns
:   -1 error, 0 discarded, 1 accepted as is, 2 accepted and trimmed

This function calls a different function depending on the method passed as input par\_TF.trimN:

- NO(0): accepts it as is, (1),
- ALL(1): accepts it as is if NO N's found (1), rejects it otherwise (0),
- ENDS(2): trims the ends and accepts it if it is longer than minL (2 if trimming, 1 if no trimming), rejects it otherwise (0),
- STRIP(3): finds the longest N-free subsequence and trims it if it is at least minL nucleotides long (2 if trimming, 1 if no N's are found), rejects it otherwise (0).

## ◆ trim\_sequenceQ()

|  |  |  |  |  |  |
| --- | --- | --- | --- | --- | --- |
| int trim\_sequenceQ | ( | Fq\_read \* | *seq* | ) |  |

trims sequence based on lowQ base callings

Parameters
:   |  |  |
    | --- | --- |
    | seq | fastq read |

Returns
:   -1 error, 0 discarded, 1 accepted as is, 2 accepted and trimmed

This function calls a different function depending on the method passed as input par\_TF.trimQ:

- NO(0): accepts is as is , (1),
- FRAC(1): accepts it if less than par\_TF.nlowQ are found (1), rejects it otherwise (0),
- ENDS(2): trims the ends and accepts it if it is longer than minL (2 if triming, 1 if no trimming), rejects it otherwise (0),
- ENDSFRAC(3): trims the ends and accepts if the remaining sequence is at least minL bases long and if it contains less than nlowQ lowQ nucleotides (2 if trimming, 1 if no trimming). Otherwise, it is rejected, (0).
- GLOBAL(4): it trims globally globleft nucleotides from the left and globright from the right, (returns 2).


---

Generated on Mon Mar 19 2018 23:42:01 for FastqPuri by  

 1.8.14
